# The gut reservoir of carbapenem-resistant Enterobacterales: from dysbiosis and colonization to infection and decolonization, with a focus on patients with hematologic malignancies - a narrative review

**DOI:** 10.3389/fcimb.2026.1939690

**Published:** 2026-08-20

**Authors:** Lorenza Putignani, Riccardo Marsiglia, Luigia Turco, Antonio Russo, Stefania Pane, Alessandra Fusco, Loris Lopetuso, Enrico Maria Trecarichi

**Affiliations:** 1 Unit of Microbiome, Bambino Gesù Children’s Hospital, Istituto di Ricovero e Cura a Carattere Scientifico (IRCCS), Rome, Italy; 2 Unit of Microbiomics, Bambino Gesù Children’s Hospital, Istituto di Ricovero e Cura a Carattere Scientifico (IRCCS), Rome, Italy; 3 Department of Life Science, Health, and Health Professions, Link Campus University, Rome, Italy; 4 Inflammatory Bowel Disease (IBD) Unit, Centro Malattie Apparato Digerente (CEMAD), Dipartimento di Scienze Mediche e Chirurgiche, Fondazione Policlinico Universitario "A. Gemelli", Istituto di Ricovero e Cura a Carattere Scientifico (IRCCS), Rome, Italy

**Keywords:** carbapenem-resistant Enterobacterales, colonization resistance, fecal microbiota transplantation, hematologic patients, microbiota-based decolonization strategies

## Abstract

Carbapenem-resistant Enterobacterales (CRE) remain among the highest-priority antimicrobial-resistant pathogens worldwide, and intestinal colonization is increasingly recognized as the key precursor of invasive infections, particularly in patients with hematological malignancies. Increasing evidence indicates that disruption of the gut microbial ecosystem, reflected in reduced diversity, depletion of beneficial anaerobic taxa, intestinal barrier dysfunction, immune dysregulation, and expansion of Enterobacterales, plays a central role in the transition from colonization to infection. Consequently, restoring colonization resistance through microbiome-targeted interventions has emerged as a promising preventive strategy. This narrative review summarizes the current evidence on the epidemiology and clinical impact of CRE colonization and infection, with particular emphasis on the ecological alterations of the gut microbiome linking gut dysbiosis to epithelial barrier dysfunction, immune dysregulation, and loss of colonization resistance to CRE persistence and invasive infection. We critically discuss both conventional and emerging decolonization approaches, including selective digestive decontamination, probiotics, prebiotics and synbiotics, fecal microbiota transplantation (FMT), bacteriophage therapy, and CRISPR-Cas-based technologies, highlighting their mechanisms of action, available clinical evidence, and current limitations. Particular attention is given to patients with hematological malignancies, in whom the clinical need for effective decolonization strategies is greatest. Although FMT currently represents the most promising microbiome-based intervention, the available evidence remains heterogeneous and largely derived from small studies. Overall, durable and standardized decolonization strategies have yet to be established, underscoring the need for well-designed multicenter randomized clinical trials to define effective microbiome-directed approaches for preventing CRE-related infections in high-risk populations.

## 1 Introduction

Infections caused by multidrug-resistant organisms (MDRO) represent a major global public health challenge and are associated with high rates of morbidity and mortality. Among these pathogens, carbapenem-resistant Enterobacterales (CRE) are classified as of critical priority by the updated 2024 WHO Bacterial Priority Pathogens List, because they have a high prevalence in several countries and are responsible for severe infections with few available treatment options and adverse outcomes, including high rates of mortality (Tumbarello et al., 2015; Maraolo et al., 2021; Sati et al., 2025). The most common mechanism of carbapenem resistance is the production of carbapenemases, a heterogeneous group of enzymes that hydrolyze most β-lactams, including carbapenems. Although both chromosomally encoded and acquired carbapenemases have been described, plasmid-encoded carbapenemases are responsible for the majority of clinically relevant and globally disseminated carbapenem resistance (Nordmann et al., 2011). Different classes of carbapenemases exist: of note, the most clinically relevant and frequently encountered include Class A enzymes, particularly *Klebsiella pneumoniae* carbapenemases (KPC), Class B metallo-β-lactamases (MBLs), such as New Delhi metallo-β-lactamase (NDM), Verona integron-encoded metallo-β-lactamase (VIM) and imipenemase-type metallo-β-lactamase (IMP); and Class D oxacillinases, mainly OXA-48-like enzymes (David et al., 2019; Hammoudi Halat and Ayoub Moubareck, 2020). Carbapenemases-producing Enterobacterales are increasingly associated with the dissemination of high-risk clonal lineages. In particular, carbapenemases-producing *E. coli* (e.g., ST38, ST131, ST167, ST361, ST405, ST410, and ST648) and *K. pneumoniae* (e.g., ST258/ST512, ST307, and ST147) are being detected with increasing frequency (Gaiarsa et al., 2015; Peirano et al., 2020; Rapid risk assessment: Increase in OXA-244 -producing Escherichia coli in the European Union/European Economic Area and the UK since 2013, first update, 2021). Notably, plasmid-encoded carbapenemase genes are also rapidly disseminated among CRE high-risk lineages (David et al., 2020).

Also, excessive activation of efflux pumps are responsible for reduction in intracellular drug concentration by extruding antibiotics (8), and porin mutations reduce antibiotic permeability (Belay et al., 2024). Given the limited treatment options for CRE infections and their significant clinical impact, CRE decolonization, especially in high-risk, immunocompromised patients, such as those suffering from hematological malignancies (HM), could be an effective preventive strategy, alongside infection control and antimicrobial stewardship measures, to reduce the negative clinical and economic impact of these infections. However, the current ESCMID-EUCIC guidelines focusing on decolonization of MDRO published in 2019 by Tacconelli et al. did not recommend routine gut decolonization of CRE carriers by using antimicrobials (such as colistin or aminoglycosides, with or without association with probiotics) because of insufficient evidence of efficacy of these procedures from available studies (including a total of 11 studies, of which two randomized and one semi-randomized controlled trials, two retrospective cohort studies, and six case series and case reports without comparators) on clinical and microbiological outcomes, and the possibility to further increase the antibiotic resistance profiles of such pathogens (Tacconelli et al., 2019). In addition, due to insufficient evidence based on nine studies conducted on multidrug-resistant Enterobacterales (of which 7 were on CRE), a recommendation for or against FMT as an MDRO decolonization strategy was not provided (12).

The aim of this narrative review was to provide a comprehensive overview of CRE colonization and infections, and alterations of the gut microbiome (GM) linking gut dysbiosis to epithelial barrier dysfunction and immune dysregulation, driving loss of colonization resistance to CRE persistence and invasive infection. Furthermore, scientific evidence on possible strategies for CRE decolonization to limit the spread of CRE strains, with a focus on HM patients, has been reviewed.

### 1.1 Epidemiology and clinical impact of CRE colonization and infection in the general population

**Figure 1:**
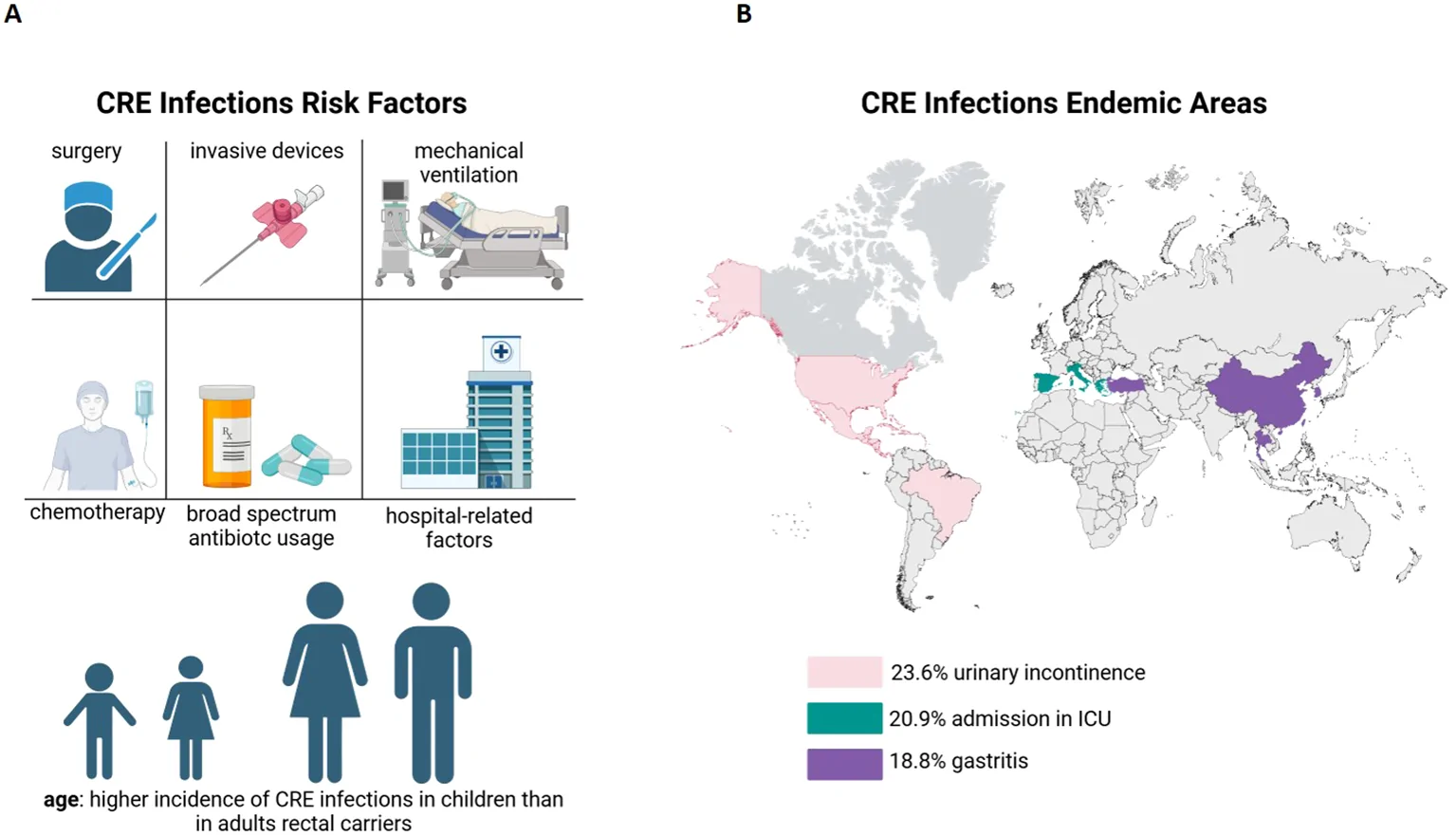
CRE epidemiology and risk factors. The left panel **(A)** shows the main risk factors for CRE infection, highlighting a correlation with age. In the right panel **(B)**, reviewed from Wei et al. (2024), endemic areas and the percentage of correlated risk factors are shown.

The most reported invasive infections caused by CRE are represented by bloodstream, respiratory and urinary with mortality rates ranging from 13.5% (for urinary tract infections) to 54.3% (for bloodstream infections), mainly related to antibiotic use (e.g., inappropriate definitive therapy), comorbidities, type and clinical severity of infection (e.g., septic shock), and hospital-related factors (such as ICU stay and mechanical ventilation) (Xu et al., 2017; Hu et al., 2022). CRE infections are associated with substantial clinical and economic consequences, including excess mortality, prolonged hospitalization, increased intensive care utilization, and limited therapeutic options. In several studies, CRE infections were associated with significantly higher attributable mortality than infections caused by carbapenem-susceptible Enterobacterales, highlighting the severe impact of resistance on patient outcomes (Viale et al., 2013; Tumbarello et al., 2015; Maraolo et al., 2021; Paniagua-García et al., 2024). Beyond their clinical burden, CRE infections impose considerable healthcare costs due to longer hospital stays, complex antimicrobial management, and infection control measures (Bartsch et al., 2017). Several risk factors have been recognized as strictly associated with CRE infections, such as older age, previous exposure to broad-spectrum antibiotics, hospital-related factors (e.g., ICU stay), previous invasive procedures (e.g., surgery), comorbidities, and previous colonization/infection by CRE (Giannella et al., 2014; Giacobbe et al., 2015; Palacios-Baena et al., 2021; Zhou et al., 2024) (**Figure 1**). In particular, with regard to previous CRE colonization, which has been highlighted as one of the most important risk factors for CRE infections, Wei et al. have recently conducted a meta-analysis of studies investigating incidence and/or risk factors of subsequent CRE infection among CRE rectal carriers (total 8188 in 24 studies) (Wei et al., 2024). Interestingly, they found a pooled incidence of CRE infections ranging from 19.8% to 23.6% across geographic regions, with ICU admission being the most important risk factor for CRE infection among adults (Wei et al., 2024).

Beyond representing a reservoir for subsequent invasive infection, intestinal colonization by CRE is increasingly recognized as both a consequence and a driver of gut microbiome (GM) disruption. The GM plays a crucial role in maintaining colonization resistance against opportunistic pathogens through mechanisms such as nutrient competition, antimicrobial metabolite production, and modulation of host immune responses (Buffie and Pamer, 2013). Conditions representing risk factors for CRE infection (e.g., exposure to broad-spectrum antibiotics and prolonged hospitalization) can profoundly alter the composition and function of the gut microbiome, resulting in loss of microbial diversity and depletion of commensal taxa that contribute to colonization resistance (Buffie and Pamer, 2013). Several studies have shown that CRE colonization is associated with a dysbiotic intestinal ecosystem characterized by reduced bacterial richness and diversity, expansion of Enterobacterales, and depletion of obligate anaerobic bacteria, including butyrate-producing taxa, which are thought to play a protective role against pathogen overgrowth (Araos et al., 2016; Gut Microbiota Alterations Associated With Colonization by Multidrug‐Resistant Organisms in ICU Patients: First Systematic Review and Meta‐Analysis - Goicea - 2026 - MicrobiologyOpen - Wiley Online Library, n.d). These microbiome alterations may facilitate persistent CRE carriage and promote progression from colonization to invasive infection, highlighting the GM as a potential target for preventive and decolonization strategies. Accordingly, increasing interest has focused on microbiota-based interventions, including fecal microbiota transplantation and other microbiome-modulating approaches, aimed at restoring colonization resistance and reducing the burden of CRE colonization and infection (Gut Microbiota Alterations Associated With Colonization by Multidrug‐Resistant Organisms in ICU Patients: First Systematic Review and Meta‐Analysis - Goicea - 2026 - MicrobiologyOpen - Wiley Online Library, n.d).

### 1.2 Epidemiology and clinical impact of CRE colonization and infection in patients with hematologic malignancies

The threat of CRE has also emerged among immunocompromised hosts, particularly patients with HM. In these patients, high-intensity chemotherapy significantly weakens their immune system, making them vulnerable to opportunistic infections, with Enterobacterales predominating, primarily due to chemotherapy-induced neutropenia and intestinal mucositis (Pouch and Satlin, 2017). In addition, the frequent use of central venous catheters and other medical devices facilitates bacterial colonization; often, long hospital stays, multiple courses of antibiotics, and other underlying conditions increase the risk of developing and spreading CRE. The increase in CRE isolates (from surveillance and clinical cultures) has been reported in recent decades, including cases of persistent bacteremia despite appropriate intravenous antibiotic treatment (Oren et al., 2013; Pagano et al., 2014; Trecarichi et al., 2015, 2023). In a large multicenter prospective study specifically addressing *K. pneumoniae* bloodstream infections in HM patients, carbapenem resistance was independently associated with significantly increased mortality, highlighting its critical clinical impact (Trecarichi et al., 2023). Mortality due to such infections in HM patients commonly exceeds 30–50% and may reach even higher rates in the presence of severe immunosuppression or delayed appropriate therapy, confirming the dramatic prognostic burden of this problem (Satlin et al., 2014; Trecarichi et al., 2016; Trecarichi and Tumbarello, 2017). Also, and particularly in this setting, previous CRE colonization has been recognized as a major factor predisposing to the development of severe infections (Trecarichi et al., 2016; Cattaneo et al., 2018).

## 2 GM ecological modifications upon CRE colonization

The human GM consists of a large microbial community inhabiting the gastrointestinal tract, including bacteria, viruses, and fungi (Fusco et al., 2017). In healthy subjects, GM performs fundamental functions in maintaining host health, contributing to the strengthening of the epithelial barrier and the maturation of the immune system. Producing short-chain fatty acids (SCFA), the GM promotes the differentiation and function of regulatory T cells (Tregs), which are crucial for maintaining immune tolerance and preventing autoimmune responses (Arpaia et al., 2013). Moreover, GM “personalizes” the repertoire of bulk secretory immunoglobulin A (IgA) antibodies in the gut (Yang et al., 2022). Increased IgA production enhances immune defense at mucosal surfaces, preventing pathogen attachment to the gut epithelium and invasion. Therefore, through the “immune education” process, GM exposes the immune system to several antigens, enhancing its ability to respond to pathogens and preventing over-reactivity to harmless antigens. Furthermore, GM is involved in the synthesis of vitamins (e.g., B1, B2, B6, B12, K, biotin, pantothenic and folic acids) and essential amino acids, in the correct absorption of minerals such as magnesium, calcium and iron, and in the acquisition of energy from food, which contribute to the maintenance of host homeostasis (Fusco et al., 2017). Moreover, the consumption of nutrients by different microbial communities (Spragge et al., 2023), along with the production of antimicrobial substances, such as lactic acid, by some beneficial bacteria (*i.e*., *Bifidobacterium*) (Belenguer et al., 2006), limits the colonization by exogenous pathogenic organisms, thus preventing their spread in the body, by a so-called “resistance to colonization” mechanism (Pickard and Núñez, 2019).

The GM ecology is characterized by remarkable dynamism; in fact, it can undergo rapid changes during the first years of life due to modulation by various factors, such as delivery mode, breastfeeding, maternal diet, and the use of antibiotics by the mother and the child (Decembrino et al., 2025). Then, GM remains fairly stable throughout adolescence and adulthood, unless perturbed by external stimuli, and its composition in adults is indeed influenced by both age and several other factors, both modifiable (e.g., diet, use of antibiotics, and stress) and non-modifiable (e.g., persistent diseases, gastrointestinal disorders, and environmental factors) (Bajinka et al., 2020; Thriene and Michels, 2023). These conditions can cause GM imbalances, leading to reduced levels of potentially beneficial bacteria and increased levels of pathogenic microbes (Falony et al., 2016; Zhernakova et al., 2016; Langdon et al., 2021). This phenomenon, called dysbiosis, commonly results in reduced GM diversity, mainly consisting of lower relative abundances of the Firmicutes and Bacteroidetes phyla, an increase in Proteobacteria relative abundance, alterations in host-microbiota mutualism, species-species network interactions, and metabolic activity and regulation (Lange et al., 2016). This loss of ecological stability can impair mucus layer integrity and weaken the epithelial gut barrier (Xie et al., 2025). This compromised barrier facilitates bacterial translocation and promotes persistent exposure of the host immune system to microbial products. At the same time, dysbiosis alters innate and adaptive immune responses by impairing macrophage, dendritic cell, and neutrophil function, reducing regulatory T-cell differentiation, and disrupting mucosal IgA-mediated immune surveillance (Levy et al., 2017; Guo et al., 2025), predisposing the host to the possible onset of several pathologies, such as inflammatory bowel diseases (e.g., Crohn’s disease), autoimmune diseases, diabetes, and obesity (Seekatz et al., 2022). Additionally, these alterations weaken colonization resistance and create a permissive intestinal ecosystem that favors pathogen colonization. Notably, dysbiosis is reported to increase the risk of colonization by CRE (and MDRO, in general), as a result of the reduction of microbial diversity; in addition, it promotes the persistence of CRE carriage, facilitating the horizontal transmission of antibiotic-resistance plasmids and, eventually, the establishment of infections (Lee et al., 2025). Notably, compared with non-colonized subjects, CRE carriers exhibit not only a reduction in GM richness but also alterations in GM composition, particularly a decrease in Bacteroidetes and Actinobacteria and an increase in Proteobacteria. At the species level, among subjects with dysbiosis, a notable rise in K. pneumoniae and Enterococcus faecium has been observed, both of which are bacterial species that cause hospital-acquired infections (Lee et al., 2024). Therefore, reverting GM dysbiosis to eubiosis may represent a new therapeutic target for many infectious diseases (Iebba et al., 2016; Bajinka et al., 2020).

### 2.1 Gut Microbiome alterations in onco-hematological patients with CRE intestinal colonization

In patients with HM, GM disruption is not exclusively a consequence of CRE colonization but rather a pre-existing and self-perpetuating condition that has a considerable impact on the risk of infectious complications and related adverse outcomes. Indeed, the GM of HM patients is already significantly altered by the underlying condition and, consequently, by the time of HM diagnosis. Studies based on *16S* rRNA gene sequencing have documented disease-specific patterns of dysbiosis across HM subtypes: in acute myeloid leukemia (AML), a significantly higher Firmicutes/Bacteroidetes ratio has been reported compared to healthy controls, along with a marked reduction in alpha-diversity indices and depletion of functionally important genera such as *Fecalibacterium*, a key butyrate-producing taxon known to exert anti-inflammatory and colonization-resistance functions (Rattanathammethee et al., 2020; Ji et al., 2025). Similarly, in chronic lymphocytic leukemia (CLL), depletion of *Bacteroidetes* and *Proteobacteria* expansion, together with loss of *Lachnospiraceae* and *Ruminococcaceae* family members, the main producers of SCFAs in the gut, and an altered Firmicutes/Bacteroidota ratio have been documented and reported to be independently associated with shorter time to first treatment and worse clinical outcomes (Faitová et al., 2022; Paziewska et al., 2024). In addition, in patients suffering from lymphoma and multiple myeloma, a reduction in microbiota composition has been demonstrated, with increased abundance of *E. coli* in the former and alterations involving *Pseudomonas aeruginosa* and *Clostridium leptum* in the latter (Guevara-Ramírez et al., 2023).

This baseline GM dysbiosis is further exacerbated by the sequential therapeutic interventions that characterize the treatment continuum for underlying hematological diseases. Cytotoxic chemotherapy directly damages the intestinal mucosal epithelium, impairs barrier integrity, and alters the luminal niche available to commensal bacteria. In addition to disrupting the GM ecosystem, these treatments profoundly impair mucosal and innate immune defenses (Nemani and Rieger, 2026). Chemotherapy-induced neutropenia reduces the first line of defense against bacterial translocation, while the damage to intestinal epithelial cells compromises barrier integrity and facilitates the passage of opportunistic pathogens into the bloodstream (Dahlgren and Lennernäs, 2023). The GM-driven immune alteration creates a biological environment that strongly favors CRE colonization. while Moreover, prophylactic and therapeutic broad-spectrum antibiotic regimens widely used in these patients to prevent and treat febrile neutropenia suppress commensals and are responsible for the selection of resistant organisms (Dumitru et al., 2025). Even more profound and prolonged (in some patients persisting over one year) GM perturbations are evident in patients undergoing HSCT, particularly allogeneic, due to more aggressive conditioning regimens and related toxicity, with severe and persistent loss of butyrate-producing bacteria, depletion of *Bifidobacterium*, and progressive dominance of pathobionts, particularly *Enterococcus* and members of the Enterobacteriaceae family (Dumitru et al., 2025).

The intersection of this compromised microbial ecosystem with CRE intestinal colonization creates a particularly dangerous clinical scenario in which CRE colonization takes on additional clinical relevance due to the high risk of progression from intestinal carriage to invasive infection, being CRE gut carriage the most important risk factor for development of subsequent bloodstream infection (Qiu et al., 2026), being prolonged neutropenia, mucosal barrier damage from high-dose chemotherapy, and impaired innate and adaptive immune responses the most important factors contributing to the translocation risk that is inherent to a dysbiotic gut dominated by CRE. Furthermore, the ratio of Enterobacteriaceae to butyrate-producing Butyricicoccaceae has been shown to highly predict bloodstream infection in HSCT recipients, with the area under the curve reaching 0.879 in one recent analysis (Yin et al., 2023). Additionally, CRE colonization itself may further promote horizontal transfer of carbapenem resistance genes among intestinal bacterial species (Lee et al., 2024), thereby worsening the ecological landscape of the gut and potentially broadening the resistome available for future pathogen emergence.

These findings suggest that in HM patients, GM dysbiosis and CRE colonization form a mutually reinforcing cycle: the pre-existing and treatment-induced GM disruption characterizing this population facilitates CRE establishment and persistence, while CRE dominance further suppresses colonization resistance, creating conditions that favor invasive, bloodstream in particular, infections. This conceptual framework strongly supports the rationale for microbiome-directed therapeutic strategies aimed at restoring eubiosis, including fecal microbiota transplantation (FMT), defined bacterial consortia, and SCFA-based interventions, as adjunctive approaches to reduce the burden of CRE colonization and prevent progression to life-threatening infection in this uniquely vulnerable patient group.

## 3 Strategies of decolonization

**Figure 2:**
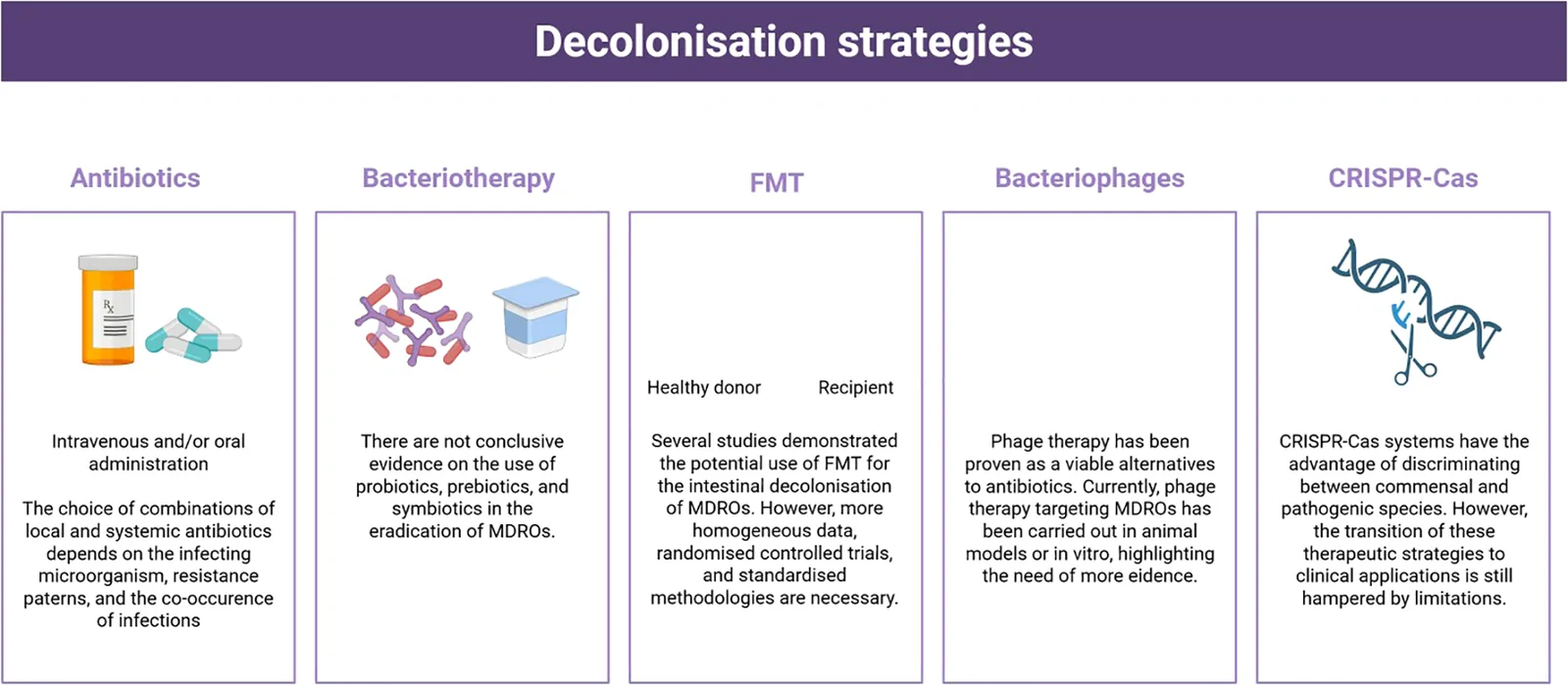
Decolonization strategies. Highlights of the main decolonization strategies reported in the last decade of literature.

Historically, MRDO decolonization strategies have focused on the use of antibiotics. However, over the last few decades, new strategies have emerged, including antibiotics, probiotics, prebiotics, synbiotics, fecal microbiota transplantation (FMT), and bacteriophage usage (**Figure 2**).

The potential benefits of decolonization are multiple, including reduced risk of MDRO infection, containment of their spread, and limitation of horizontal gene transfer (Catho and Huttner, 2019). Among MDRO, CRE are among the most relevant targets for intestinal decolonization, because the gastrointestinal tract is the main reservoir for CRE persistence, dissemination, and subsequent invasive infections. In this setting, CRE decolonization is not only the removal of a resistant strain, but also a possible strategy to restore gut colonization resistance and reduce the intestinal spread of carbapenem-resistance genes (55).

A recent systematic review and meta-analysis showed that selective digestive decontamination (SSD) and FMT are the most investigated strategies for MDR (including carbapenem-resistant) Enterobacterales, with mainly short-term benefits, while long-term efficacy and standardized protocols still remain uncertain (Zhang et al., 2024). The following (**Figure 2**) are the main methodologies used to date as strategies for intestinal decolonization of MDRO CRE in particular.

### 3.1 Antibiotics

The choice of effective antimicrobial therapy for CRE infections is often difficult, as susceptibility patterns are unpredictable and depend on the specific clinical context; therefore, antibiotic selection must be guided by the results of specific *in vitro* tests (Sheu et al., 2019) traditionally relied on oral and/or intravenous antibiotics,. Several novel agents active against CRE have recently entered clinical practice, including plazomicin, eravacycline, cefiderocol, and new β-lactamase inhibitors, as well as combinations of ceftazidime-avibactam, aztreonam-avibactam, and meropenem-vaborbactam, replacing therapeutic regimens which were traditionally used previously, including in particular colistin-based combination regimens (e.g., polymyxin, fosfomycin, high-dose tigecycline) and double-carbapenem therapy (Dong et al., 2020; Rodríguez Feria et al., 2025) (Suay-García and Pérez-Gracia, 2019; Yusuf et al., 2021).

Broad-spectrum intravenous antibiotics have been combined with selective digestive tract decontamination (SDD), which involves administering non-absorbable antibiotics into the stomach (Roson-Calero et al., 2023). SDD has been used as a prophylactic intervention for several decades to reduce or eliminate gut colonization by pathogenic agents, thereby preserving beneficial endogenous flora, to reduce morbidity and mortality in high-risk settings, primarily in the ICU (Hammond et al., 2022; van Doorn-Schepens et al., 2022). The choice of antibiotic drugs to be used, whether systemically or locally, depends on the infecting microorganism, resistance patterns, and the co-occurrence of infections.

Antibiotics with poor enteral absorption, including neomycin sulphate, colistin sulphate, paromomycin, and gentamicin, have been used topically for SDD. In CRE carriers, SDD has been proposed especially in high-risk settings, such as HM patients, since CRE colonization may precede severe infections during neutropenia, chemotherapy, immunotherapy, or hematopoietic stem cell transplantation (HSCT) (56). In a recent meta-analysis, one of the analyses including three RCTs, SDD showed a significantly better decolonization effect than controls at the end of treatment and a higher decolonization rate at one month with an OR of 4.01 (95%CI 1.88-8.56), although the effect appeared mainly short-term and its long-term durability remains unclear (Zhang et al., 2024). A further systematic review and meta-analysis reported that SDD significantly increased CRE clearance compared with controls, including placebo or study arms without a decolonization measure, but the durability of this effect was limited, and antimicrobial resistance to intervention antibiotics was reported as a relevant concern (McCafferty et al., 2026).

Several studies have highlighted the limitations of using systemic or non-absorbable antibiotics in eradicating MDRO from the intestinal tract (Rieg et al., 2015; Bar-Yoseph et al., 2016). These include the risk of increased antibiotic resistance, especially to polymyxins and aminoglycosides used for decolonization, and the short duration of bacterial clearance after decolonization (Huttner et al., 2013; Katchman et al., 2014), leading to the so-called “rebound effect”, namely the re-emergence or rapid recolonization of MDRO following an initial period of successful suppression (Oostdijk et al., 2010). Lastours et al. confirmed, in a randomized, multicenter clinical trial, the risk of secondary colistin resistance after its administration for SDD and clarified the molecular mechanisms underlying this phenomenon (de Lastours et al., 2020).

Furthermore, the recent study by Tancharoen et al. evaluated the efficacy and safety of neomycin for CRE decolonization (Tancharoen et al., 2024) and reported temporary efficacy against CRE colonization, which was highest after 9 days of treatment but waned after 16 days.

Other important concerns have also been raised regarding the potential negative effects of antibiotic use for decolonization. In fact, antibiotic use can alter the patient’s microbiota composition and increase the incidence of dysbiosis-related diseases (Halaby et al., 2013). This aspect is particularly relevant for CRE, because broad-spectrum antibiotics can alter the intestinal ecological niche in favor of CRE expansion. Antibiotics may reduce important commensal families, enrich nutrients that CRE can use for growth, and deplete microbial metabolites that normally inhibit CRE proliferation (Yip et al., 2023). Moreover, antibiotic exposure may promote horizontal transfer of carbapenem-resistance genes in the gut microbiome. In an experimental model, ampicillin and amoxicillin enriched both carbapenem-resistant *K. pneumoniae* and susceptible *E. coli*, favoring the transmission of a blaNDM-1-bearing plasmid, mainly through Proteobacteria expansion and Firmicutes suppression (Ye et al., 2019).

For these reasons, the ESCMID-EUCIC1 clinical guidelines on the decolonization of multidrug-resistant Gram-negative bacteria (Tacconelli et al., 2019) do not currently recommend decolonization as a routine procedure and instead call for more clinical trials involving high-risk patients for whom temporary suppression would be clinically significant. Indeed, decolonization practices must be integrated into antibiotic stewardship, which promotes the prudent use of antibiotics by reserving them for infections rather than for simple colonization (Guidelines for the Prevention and Control of Carbapenem-Resistant Enterobacteriaceae, Acinetobacter Baumannii and Pseudomonas Aeruginosa in Health Care Facilities, 2017).

### 3.2 CRISPR-Cas based technology

Among emerging innovative strategies for gut decolonization of MDRO, Clustered Regularly Interspaced Short Palindromic Repeats – associated protein (CRISPR-Cas) systems have been reported to specifically and precisely modify the gut microbiome resistome, serving as an alternative to broad-spectrum antimicrobials and protecting against invaders via adaptive and heritable immunity (Bikard et al., 2014; Yosef et al., 2015; Hsu et al., 2020; Neil et al., 2021; Reuter et al., 2021; Brödel et al., 2022).

CRISPR-Cas systems have the advantage of effectively discriminating between commensal and pathogenic species, as they can be programmed to target specific genes or inhibit their expression.

One of the main challenges of this therapeutic approach is designing the most efficient delivery system. Current strategies include the use of polymer nanocomposites, microparticles, phages, or conjugated bacteria (Fagen et al., 2017). Neil et al. recently proposed the COP system, represented by an engineered *E. coli* strain, Nissle 1917, harboring the CRISPR-Cas9 system on a conjugative plasmid, TP114, which reduced the target population by over 99.9% in a mouse model (Neil et al., 2021). Engineered bacteriophages, called CR-phages, can also be used as vectors for the CRISPR system. These detect and bind receptors on the outer membrane or capsule of the target bacterium, and insert CRISPR-conjugated genetic elements into the pathogen’s cytoplasm (Nath et al., 2022). Several studies have also demonstrated the use of phagemids and temperate phages active against ESKAPE species, including *E. coli* (Bikard et al., 2014; Yosef et al., 2015) and *K. pneumoniae* (Nath et al., 2022). In one study, in particular, the role of CRISPR-Cas was demonstrated in combating Carbapenem-resistant *K. pneumoniae* (Jurić et al., 2025, 2025). CRISPR-Cas systems were found in 15.8% of the 400 clinical *K. pneumoniae* isolates analyzed. Isolates lacking the CRISPR-Cas immune defense exhibited higher rates of multidrug resistance and resistance to agents such as imipenem/relebactam, indicating that the absence of CRISPR-Cas may allow bacteria to more readily acquire resistance-carrying plasmids (Jurić et al., 2025).

Combination therapies are currently being designed to broaden the available strategies and effectively mobilize all CRISPR elements into the target cell. However, although significant progress has been made in this field, the transition of these therapeutic strategies to clinical application is still hampered by some problems, such as the *in vivo* biocontainment of the system’s transporters, the still narrow spectrum of activity in the case of bacteriophages, and the low conjugation ratios (Fagen et al., 2017; Dong et al., 2019; Reuter et al., 2021; Brödel et al., 2022; Murugaiyan et al., 2022; Nath et al., 2022). Indeed, to date CRISPR-Cas-based approaches remain mainly experimental for CRE intestinal decolonization and are actually considered as future microbiome-editing strategies to be further investigated (55).

### 3.3 Bacteriophages

Bacteriophages are the most abundant bacterial predators (Tacconelli et al., 2014) and have been used in the former Soviet Union countries since the 1920s in standard medical practice, as they are not subject to medical prescription (Salmond and Fineran, 2015). In Western countries, the development of cutting-edge technologies has sparked renewed interest in phage therapy research to eradicate MDRO (Lin et al., 2017; Baral, 2023).

Phage therapy and phage-derived enzymes have been demonstrated to be viable alternatives to antibiotics, with preclinical and clinical studies supporting their effectiveness in reducing bacterial load, eradicating biofilms, and accelerating wound healing (Yang et al., 2025). Moreover, phages are suitable for patients with weak immune systems because they have no toxic effects or immunological complications (Abedon and Thomas-Abedon, 2010).

One of the main advantages of this therapeutic approach is the ability to selectively target phages against MDRO, thanks to their specificity for host bacteria; this feature also allows preservation of the remaining intestinal microbiota. Currently, most research on phage therapy for MDRO gut decolonization has been carried out in animal models or *in vitro* (Galtier et al., 2016; Javaudin et al., 2021; Fang et al., 2022). However, in the context of intestinal CRE carriage, the available evidence is still mainly preliminary, and phage therapy represents a strategy that is still largely under development and the available data are still scarce and also conflicting. In some studies using murine models, a reduction in intestinal *E. coli* load following bacteriophage therapy has been demonstrated and the eradication of a strain carbapenemase-producing *K. pneumonaie* was obtained in a 57-year-old patient with Crohn’s disease and recurrent UTI (Galtier et al., 2016, 2017; Cepko et al., 2020; Corbellino et al., 2020). Conversely, other studies have shown that it is difficult to reduce the gut carriage of *E. coli in vivo* using phages (Chibani-Chennoufi et al., 2004; Weiss et al., 2009; Tao et al., 2021).

In general, phage therapy currently faces numerous limitations, such as the specificity of phages against MDRO, and the lack of data derived from RCTs investigating quality, safety, microbiological, clinical, and epidemiological efficacy, route of administration, dose dependency, intestinal microbiota, and patient age, as well as the possible secretion of digestive compounds that influence stomach acidity (Dąbrowska, 2019; Roson-Calero et al., 2023). Furthermore, phages can also transfer unwanted genes and interact with the host’s immune system and microbiome (Lin et al., 2017; Roach and Debarbieux, 2017; Arena et al., 2018; Harper, 2018; Dąbrowska, 2019; Brives and Pourraz, 2020; Herridge et al., 2020).

### 3.4 Bacteriotherapy: probiotics, prebiotics, and synbiotics

The WHO defines probiotics as “live microorganisms which, when administered in adequate amounts, confer a health benefit on the host” (Hill et al., 2014). Over the past decade, scientific and public interest in administering these microorganisms for the treatment and prevention of various diseases has grown significantly. Their beneficial effects on the intestine include improved digestion, prevention of antibiotic-associated diarrhea, and relief of irritable bowel syndrome symptoms (Doron and Gorbach, 2006). Since their introduction as dietary supplements to modulate the composition of the intestinal microbiota, few studies have reported links between their intake and changes in the host resistome (Newman and Arshad, 2020). In addition to reshaping microbial composition, probiotics may exert beneficial effects by modulating host immune responses and reinforcing intestinal barrier function, enhancing tight junction integrity, stimulating mucus production, exerting strain-dependent anti-inflammatory properties, promoting the production of microbiota-derived metabolites, and regulating both innate and adaptive immune responses (Guo et al., 2025). Unfortunately, to date, knowledge gained from various RCTs has not demonstrated statistical significance in eradicating MDRO from the intestinal tract compared with placebo (Karbalaei and Keikha, 2022). CRE colonization has been associated with intestinal dysbiosis, reduced microbial diversity, and enrichment of Enterobacteriaceae, along with the depletion of anaerobic commensals (Korach-Rechtman et al., 2020). Moreover, in critically ill patients, CRE colonization induces increased Proteobacteria and reduced Bacteroidetes, together with alterations in SCFA production and other predicted metabolic pathways, suggesting that the microbiota metabolism may influence CRE persistence in the gut (23). These aspects could provide an interesting rationale for the use of probiotics, synbiotics, and prebiotics in CRE-colonized patients.

Even the 2019 ESCMID/EUCIC guidelines do not recognize sufficient evidence to support the use of probiotics for this purpose, also because many aspects of this approach, such as frequency, dosage, or route of administration, have not been adequately clarified by well-conducted clinical studies. Regarding CRE, clinical evidence supporting the use of probiotics for intestinal decolonization remains insufficient. In a recent systematic review and meta-analysis, too few studies evaluating probiotics for extended-spectrum beta-lactamases (ESBL) producing Enterobacterales (ESBL-E)/CRE decolonization were included, so a quantitative meta-analysis could not be performed (Zhang et al., 2024).

However, some studies have reported encouraging results. Some commensal or probiotic bacteria have been reported to interfere with antibiotic-resistant Enterobacterales; the supernatant of *Clostridium butyricum*, *Enterococcus faecium*, and *Lactobacillus plantarum* suppressed the growth of ESBL-producing *E. coli* (ESBL-Ec) and CRE in a dose-dependent manner, although these *in vitro* results cannot be directly translated into clinical CRE eradication (Kunishima et al., 2019). Microbiota-derived metabolites may also contribute to CRE colonization resistance. Butyrate, a metabolite associated with a healthy GM, was shown to inhibit the growth of carbapenem-resistant *E. coli*, with effects varying by resistance mechanism: porin-deficient isolates were more sensitive to butyrate than carbapenemase-producing isolates (Happ et al., 2024).

In 2022, Guitor A.K. et al. showed the potential activity of *Bifidobacterium* spp. and *Lactobacillus* spp. in replacing antimicrobial resistance genes (ARGs) from Enterobacterales in the intestine of preterm infants (Guitor et al., 2022). Conversely, Boysen et al (Dall et al., 2019). found no positive effect of using this same species in preventing and reducing the risk of acquiring ESBL-E colonization in travelers. Another RCT tested the probiotic Vivomixx^®^, a combination of 8 probiotic strains, in patients chronically carrying intestinal ESBL-E, and found that after 2 months of treatment, only 12.5% of subjects taking the probiotic had achieved the eradication goal, indicating insufficient efficacy compared with placebo (Ljungquist et al., 2020).

Next-generation probiotic strains have been recently identified. Preclinical studies have been conducted on the species *A. muciniphila*, *Bacteroides uniformis*, *F. prausnitzii*, or the *Clostridium* groups IV, XIVa, and XVIII (Patel and DuPont, 2015). Moreover, in 2023, Deng et al. developed nature-inspired artificial probiotics based on covalent organic frameworks, dispersed within artificial enzymes, as a novel strategy to modulate GM. While evidence is currently limited to preclinical models of intestinal inflammation, their application to MDRO carriage and infections has been proposed as a potential microbiota-based intervention tool (Deng et al., 2023). In one screening study, five candidates out of 57 *Lactobacillus* spp. strains (LUC0180, LUC0219, LYC0289, LYC0413, and LYC1031) inhibited carbapenem-resistant *E. coli* (CRE316) and *K. pneumoniae* (CRE632) (Chen et al., 2019). In addition to these strains, other species, including *Bifidobacterium longum*, *Lactiplantibacillus plantarum*, and *Lacticaseibacillus rhamnosus*, have also demonstrated notable antimicrobial activity against CRE (Chornchoem et al., 2025). Clinically, a retrospective analysis of ICU patients showed that among 474 individuals receiving probiotics, the incidence of new CRE colonization was significantly reduced, with only 13 patients developing it, compared with a markedly higher rate in the control group (Lee et al., 2023; Zhang et al., 2025).

Postbiotics represent an important microbiota-targeted therapeutic approach, acting as bioactive microbiota-derived metabolites and exerting beneficial effects on host cells and tissue. They include inactivated cell-free supernatants, exopolysaccharides, cell wall components (e.g., lipoteichoic acids), bacterial lysates, secreted proteins (e.g., lactocepin, the p75 molecule), metabolites, vitamins (e.g., vitamin B, vitamin K), carbohydrates (e.g., teichoic acids), and enzymes (e.g., glutathione peroxidase and peroxide dismutase) (Rafique et al., 2023). A notable example includes SCFAs, metabolites that exert anti-inflammatory effects by inhibiting the activation of NF-κB and the production of proinflammatory cytokines (Omenetti and Pizarro, 2015). They can also promote the differentiation of Treg cells and suppress that of Th17 cells (Zhou et al., 2022). Unlike probiotics, postbiotics are molecules rather than living organisms, offering advantages such as easier standardization, storage, and reduced risk of carrying virulence factors or antibiotic resistance genes, which make them safer (Imperial and Ibana, 2016). Although evidence in CRE decolonization is currently scarce, recently postbiotics derived from *Lactobacillus* spp. have been reported to have inhibitory effects on the growth of multidrug-resistant *K. pneumoniae* when applied in combination with low-dose antibiotics (Aghamohammad et al., 2025). These findings highlight the potential of postbiotics as adjunctive therapeutic agents in the battle against CRE while avoiding some of the safety concerns associated with live bacterial administration.

The other main approach for bacteriotherapy is prebiotics, which offer an advantage over probiotics since they do not contain living organisms. Prebiotics are non-digestible products, including fructo-oligosaccharides, inulin, and lactulose, naturally produced by the commensal microbiota and fermented into SCFA, especially by Lactobacilli and Bifidobacteria, providing a favorable growth environment and increasing the biodiversity of intestinal commensals (Roson-Calero et al., 2023). Unfortunately, fewer studies have confirmed the efficacy of prebiotics in eradicating MDROs. Hence, in a pilot trial, the effect of inulin on gut SCFA-producing bacteria in ICU patients was evaluated, with no significant trends in efficacy (Freedberg et al., 2020).

Finally, the combined administration of probiotics (including includes *Bifidobacterium* species, Lactobacilli, and *Saccharomyces boulardii*) and prebiotics (including often an oligosaccharide, such as fructo-oligosaccharide or inulin), which is defined as synbiotic treatment (Kanamori et al., 2003; Pandey et al., 2015; Asahara et al., 2016; Phavichitr et al., 2021), has been reported to reduce *Clostridium difficile* colonization in healthy infants, and to have a beneficial effect in controlling MDRO infection in murine models (117,119,120). One study investigated *in vitro* and in preclinical murine models the inhibitory effect of *Lactobacillus* spp. with prebiotics against KPC-2-producing *K. pneumoniae* (Tang et al., 2022). The authors found that lactulose/isomalto-oligosaccharide/inulin and fructo-oligosaccharide can enhance the inhibitory effect of *Lactobacillus* strains (LYC1154, LYC1322, and LYC1511) against KPC001 (Tang et al., 2022).

Looking beyond classic microbiome-based interventions, advanced strategies are expected to move toward precision microbiome therapeutics. Engineered probiotics have developed rapidly in recent years. They leverage gene editing, such as CRISPR, to perform programmable therapeutic tasks by modifying original probiotics. To date, several engineered probiotics have been studied for their efficacy in counteracting bacterial infections. An engineered probiotic based on *E. coli* Nissle 1917 was shown to specifically target and kill vancomycin-resistant Enterococci (Geldart et al., 2018). The same *E. coli* strain was also engineered to specifically kill pathogens by detecting the autoinducer N-acyl homoserine lactone (AHL) of *P. aeruginosa* and releasing antibiotics and anti-biofilm enzymes (Hwang et al., 2017).

In addition to this, defined microbial consortia were also reported to restore colonization resistance, especially in *C. clostridium* infections (Collins and Auchtung, 2017), paving the way for new promising bacteriotherapy strategies to combat CRE colonization.

Overall, available evidence on bacteriotherapy remains insufficient to recommend probiotics, prebiotics, and synbiotics as a routine CRE decolonization strategy, mainly because of heterogeneous study designs, limited sample sizes, variability in formulations, and inconsistent clinical outcomes.

### 3.5 Fecal microbiota transplantation

FMT is a technique that directly alters GM composition to restore a healthy microbiome, based on the administration of a solution of fecal material, either frozen, encapsulated, or fresh, from a healthy donor to a recipient. The first phase of the process involves a thorough screening procedure to identify a suitable donor who is mostly healthy and unrelated (Cammarota et al., 2019). The donor is required to complete a questionnaire regarding their family medical history and current exposure to any medications. Additionally, the donor must undergo a series of laboratory tests to ensure the absence of pathogens and communicable diseases. Restoring GM balance and modulating immune responses through FMT has emerged as a promising therapeutic strategy to improve disease outcomes. Indeed, FMT reshapes the GM and modulates immune responses, enhancing Tregs, increasing *Bifidobacterium* abundance, and suppressing pro-inflammatory Th1 and Th17 responses (Guo et al., 2025).

FMT is now recommended by international clinical guidelines as a treatment for active and recurrent *C. difficile* infections (CDI) refractory to all other treatments in cases of severe dysbiosis (Debast et al., 2014; McDonald et al., 2018; Cammarota et al., 2019). Indeed, the high therapeutic efficacy (between 90% and 100%) of FMT in this condition has been widely demonstrated (Kelly et al., 2015; Ianiro et al., 2018), as well as its good safety profile, although rare episodes of bacteremia have been reported (Baxter and Colville, 2016). The mechanism by which FMT replaces *C. difficile* and reconstitutes the intestinal microbiota is unknown; however, recent studies have demonstrated that it can reduce the abundance and expression of antibiotic resistance genes in adult patients with recurrent CDI (129,130), and it is also effective in the pediatric population (Marsiglia et al., 2024).

FMT has also been described as a promising therapy for eradicating other MDRO, including CRE, due to a complex interaction between the GM and its host that affects important immune functions and helps maintain intestinal homeostasis (Cani and Delzenne, 2009; Hsiao et al., 2013; Singh et al., 2018; Baek et al., 2023; Alaeddin et al., 2025). Among microbiota-based strategies, FMT is the most studied approach for CRE intestinal decolonization. Its rationale is to restore a more diverse and balanced microbial ecosystem, increasing colonization resistance and reducing the intestinal reservoir of antibiotic-resistant Enterobacterales (44).

Recent meta-analyses suggest that FMT may have a favorable effect on CRE decolonization, although available studies are heterogeneous. In one systematic review and meta-analysis, FMT significantly improved CRE clearance compared with controls (McCafferty et al., 2026). In another systematic review and meta-analysis, FMT showed a significantly higher rate of decolonization compared with one month of CRE infection treatment, while long-term efficacy and protocol standardization remained unclear (Zhang et al., 2024). Oral capsulized FMT has also been evaluated for the eradication of CRE colonization, supporting the concept that FMT may act by remodeling the intestinal microbiome and favoring a more robust ecological state that can prevent CRE carriage (Bar-Yoseph et al., 2021). A pilot study evaluated longitudinal dynamics of the bacteriome and virome in three FMT recipients who were successfully decolonized from CRE. After FMT, the bacterial microbiota shifted toward a donor-like community, while changes in phage populations were observed alongside a reduction in CRE species, suggesting that both the bacteriome and virome may contribute to the effects of FMT (Liu et al., 2022). In CRE carriers undergoing FMT, GM changes were also evaluated across different decolonization periods (40%, 50%, and 90% of patients decolonized within 1, 3, and 5 months after initial FMT), showing that FMT-induced CRE clearance was associated with changes in GM composition (Lee et al., 2021). In a prospective comparative trial including patients colonized with CRE and/or VRE, FMT showed a higher overall negative conversion rate at three months compared to controls, while FMT-induced CRE clearance showed a favorable trend but was not always statistically significant (Shin et al., 2022). Gargiullo et al. reviewed results from 23 analyzed reports in which 142 patients underwent FMT for MDR intestinal decolonization: 31.6% of treated patients received FMT via the nasoduodenal route, 25.3% via the nasogastric route, and 16.8% via oral capsules. The most commonly isolated MDR intestinal bacteria pre-FMT were CRE and VRE; MDRO in other sites, including *P. aeruginosa*, MRSA, and *Acinetobacter*, were also reported. Fecal samples and rectal swabs from 77.5% of patients showed microbiological clearance of MDROs (Gargiullo et al., 2019). From the 23 reports analyzed, one serious adverse event was reported, resulting in liver cirrhosis and the patient was hospitalized 2 weeks after FMT for an episode of encephalopathy (Gargiullo et al., 2019). Another review was conducted by Yoon et al., who reported an MDRO-decolonization efficacy of 70.3% in a cohort of 121 patients who underwent FMT for 146 MDRO (including CRE) across 20 articles (Yoon et al., 2019).

Dinh et al. conducted a multi-center trial on 17 patients with CRE or VRE digestive tract colonization who received FMT between January 2015 and April 2017 (Dinh et al., 2018). One week after FMT, three of eight patients were free of CRE colonization, and three of nine patients were free of VRE colonization. After three months, four of eight patients were free of CRE colonization, and seven of eight patients were free of VRE colonization (Dinh et al., 2018). In the FERARO feasibility randomized controlled trial, encapsulated lyophilized FMT was evaluated in patients with ESBL-E or CRE gastrointestinal carriage after invasive infection. Colonization decreased over time in both arms, and FMT also increased microbiome diversity and microbiome-based health measures, thereby enhancing colonization resistance (Merrick et al., 2025).

Despite the promising data reported above, more recent studies have, however, provided conflicting evidence. In a prospective multicenter study of CRE-colonized patients, complete CRE clearance was observed in 20% of patients two weeks after a single FMT dose and in 40% at three months; however, comparison with a control group showed a similar decolonization rate, indicating that spontaneous clearance must be considered; in addition, responders had lower CRE relative abundance before FMT and higher bacterial richness and alpha diversity after FMT (Davido et al., 2024).

In conclusion, more robust and homogeneous data from ongoing RCTs are needed to confirm the benefit of this therapeutic approach compared with no treatment (Tavoukjian, 2019). Several factors should be also evaluated, including i) the most effective route of administration (oral, colonic, or enteric); ii) sample preservation (frozen or fresh stool); the ideal donor (family member or autologous, the prevalence of one or more taxa, and the phylogenetic diversity); iii) methods for donor identification; iv) antibiotic-based GM depletion before FMT (Manges et al., 2016; Davido et al., 2018; Huttner et al., 2019; Wilson et al., 2019). Moreover, spontaneous decolonization may occur and should be considered when interpreting FMT efficacy (Bilsen et al., 2022). For all these reasons, the ESCMID guidelines do not make any recommendations for or against the use of FMT for intestinal decolonization of MDRO (Righi et al., 2024).

## 4 Current evidence of CRE decolonization strategies in patients with hematological malignancies

Despite the urgency of their clinical impact, evidence for effective MDRO decolonization strategies in HM patients, particularly for CRE, remains limited and largely derived from small, heterogeneous studies (**Table 1**). SDD with non-absorbable oral antibiotics is one of the most extensively studied approaches (De Abreu Luís and Mimoso Santos, 2025). In a seminal single-center pilot study conducted in a haemato-oncology/HSCT unit following an outbreak of carbapenem-resistant *K. pneumoniae*, Zuckerman et al. administered oral gentamicin (80 mg four times daily) to 15 rectal CRKP carriers, achieving an eradication rate of 66% and cessation of persistent bacteremia in 62.5% of treated patients (Zuckerman et al., 2011).

**Table 1:** Overview of clinical studies evaluating CRE decolonization strategies in patients with hematological malignancies, including study design, patient population, decolonization approach, and clinical outcomes.

Author, year	Country	Enrollment period	Design of study	Patient setting	Pediatric/adult	Decontamination target/objective	CRE proportion in study population	Type of decontamination	Outcome	Observation time
Selective digestive decontamination										
Zuckerman et al., 2011	Israel	September 2008–January 2009	Single-center pilot study/observational experience	Hematology-oncology and BMT unit; patients undergoing intensive chemotherapy or SCT and colonized by CRKP	Adults	Eradication of gastrointestinal CRKP carrier state; prevention of infection and nosocomial spread	15/15, 100% CRKP	Oral gentamicin 80 mg q.i.d. until eradication; continued during HSCT period in selected high-risk patients	CRKP eradication in 10/15 patients; persistent bacteremia resolved in 5/8 bacteremia patients; no significant side effects; no emergence of gentamicin resistance	Median persistence of eradication 9 months, range 2–10 months
Oren et al., 2013	Israel	June 2009–May 2011	Semi-randomized prospective controlled trial	Tertiary-care hospital; consecutive hospitalized CRE carriers, including hematology/BMT and other inpatients	Adults	Eradication of gastrointestinal CRE colonization; prevention of transmission and clinical infection	152/152, 100% CRE by inclusion criteria; treated group 50/50 and control group 102/102	Oral non-absorbable antibiotics according to susceptibility: gentamicin, colistin, or both	Eradication: gentamicin 42%, colistin 50%, gentamicin + colistin 37.5%; all higher than spontaneous eradication in controls, 7%; no significant adverse effects	Median treatment/follow-up 33 days in treated patients; median follow-up 140 days in controls
Lambelet et al., 2017	Italy	Not reported	Prospective single-center experience	Hematology ward; hematologic patients with KPC-Kp-positive rectal swabs	Adults	Gut decontamination from KPC-producing K. pneumoniae before immunosuppressive therapy, SCT, or to prevent post-chemotherapy sepsis	14/14, 100% KPC-Kp	Oral gentamicin 80 mg q.i.d. for 7–25 days	Overall decontamination 10/14, 71%; 9/10, 90%, among patients receiving oral gentamicin only; no new gentamicin-resistant KPC-Kp strains	Follow-up 3 months after proven decontamination
Stoma et al., 2018	Belarus	November 2016–October 2017	Randomized controlled trial, non-blind, parallel assignment 1:1	Tertiary hematology center; patients with hematological malignancies and MDR/XDR Gram-negative intestinal colonization	Adults	Selective intestinal decolonization to reduce MDR/XDR Gram-negative carriage and prevent BSI	18/62, 29.0% CRE, calculated as carbapenem-resistant *K. pneumoniae* or *E. coli* at baseline; other carbapenem-resistant pathogens included *A. baumannii* and *P. aeruginosa*	Oral colistin for 14 days vs watch-and-wait control	Significant short-term reduction in colonization at day 14: 61.3% vs 32.3%; no sustained difference at day 21 post-treatment; lower BSI incidence in first 30 days, but no significant benefit over 90 days; no serious adverse effects or increase in colistin resistance	Primary assessment day 21 post-treatment; BSI observation 90 days
Fecal microbiota transplantation										
Su et al., 2021	China	Single patient treated in 2018 (allo-HSCT performed in March 2017; FMT July-August 2018)	Case report	Allogeneic hematopoietic stem cell transplant (allo-HSCT) recipient with acute myeloid leukemia (AML), recurrent carbapenem-resistant *Klebsiella pneumoniae* colonization/infection	Adult	Eradication of intestinal carbapenem-resistant Enterobacteriaceae (CRE) colonization to prevent recurrent infection	100% (1/1 patient colonized with CRE/CRKP)	Tandem fecal microbiota transplantation (FMT): two FMT cycles, each consisting of three nasoduodenal infusions administered every other day (total 6 FMT procedures)	Successful eradication of CRE colonization; stool cultures remained CRE-negative; no treatment-related adverse events; microbiota diversity increased and gut microbiota composition partially restored	26 months follow-up after FMT; patient remained clinically stable with persistent CRE-negative stool cultures
Battipaglia et al., 2019	France	2014–2017	Retrospective single-center study	Hematologic malignancy patients undergoing FMT before or after allo-HSCT for MDRB colonization or infection	Adults	MDRB decolonization, mainly to reduce infectious complications and facilitate transplant/clinical management	4/10, 40% CPE/CRE; CPE included E. coli, *Citrobacter freundii* and *K. pneumoniae*; one patient was colonized by three different CPE	FMT from healthy related or unrelated donors; delivery by enema or nasogastric tube; repeat FMT in selected failures	Decolonization in 7/10 patients; persistent decolonization at last follow-up in 6/10; mild gastrointestinal adverse events; one case of grade III gut acute GVHD after FMT	Median follow-up 13 months, range 4–40; significant infections assessed during the first 90 days after FMT
Masetti et al., 2025	Italy	November 2018–November 2024	Retrospective multicenter real-world study	Paediatric allo-HCT units in Rome, Bologna and Padua; paediatric patients colonized by MDR bacteria receiving FMT before or after allo-HCT	Paediatric, <21 years	Safety and efficacy of FMT for MDR decolonization; analysis of factors associated with decolonization efficacy	Exact CRE proportion not reported in the main manuscript; 14/22, 64%, were colonized by VIM-producing strains, but species-level CRE/CPE classification is reported in supplementary material	FMT via upper gastrointestinal tract: EGDS, naso-jejunal tube or PEJ; unrelated donors; mostly frozen material; multiple infusions in selected patients	Decolonization 77% at 1 week and 52% at 6 weeks; no severe FMT-related adverse events; more than one FMT associated with higher 6-week decolonization; no MDR-BSI among patients decolonized at 6 weeks	Decolonization assessed at 1 and 6 weeks after first FMT; infectious outcomes described during early post-HCT follow-up
Bilinski et al., 2017	Poland	February 2015–July 2016	Prospective single-center study	Department of Hematology, Oncology and Internal Diseases; patients with blood disorders and ARB gut colonization	Adults	Complete eradication of ARB from the gastrointestinal tract	Patient-level CRE proportion could not be clearly derived because several patients carried multiple ARB strains. CRE/CPE strain entries included *K. pneumoniae* NDM-1, other carbapenem-resistant *K. pneumoniae*, carbapenem-resistant *Enterobacter cloacae* and *E. coli* OXA-48	FMT from unrelated healthy donors; intraduodenally administration via nasoduodenal tube	Complete ARB decolonization in 15/25 FMTs at 1 month and in 15/20 patients overall; partial decolonization in 20/25 FMTs; no severe adverse events	Rectal swabs at 1 week, 1 month and 6 months; median follow-up 187 days, range 9–482

ARB, antibiotic-resistant bacteria; allo-HCT, allogeneic hematopoietic cell transplantation; allo-HSCT, allogeneic hematopoietic stem cell transplantation; BMT, bone marrow transplantation; BSI, bloodstream infection; CDI, *Clostridioides difficile* infection; COL, colistin; CRE, carbapenem-resistant Enterobacterales/Enterobacteriaceae; CRKP, carbapenem-resistant *Klebsiella pneumoniae*; CTIWP, Cellular Therapy and Immunobiology Working Party; EGDS, esophagogastroduodenoscopy; FMT, fecal microbiota transplantation; GI, gastrointestinal; GM, gentamicin; GVHD, graft-versus-host disease; HCT, hematopoietic cell transplantation; HSCT, hematopoietic stem cell transplantation; KPC-Kp, *Klebsiella pneumoniae* carbapenemase-producing *Klebsiella pneumoniae*; MDR, multidrug-resistant; MDRB, multidrug-resistant bacteria; NJT, naso-jejunal tube; PEJ, percutaneous endoscopic jejunostomy; q.i.d., four times daily; RCT, randomized controlled trial; SCT, stem cell transplantation; SDD, selective digestive decontamination; VRE, vancomycin-resistant enterococci; XDR, extensively drug-resistant.

Based on this experience, the same group conducted a larger semi randomized prospective controlled trial enrolling 152 CRE carriers over a 24-month period, of whom 50 received tailored non-absorbable antibiotic therapy guided by individual susceptibility profiles, oral gentamicin, colistin, or their combination, while 102 served as untreated controls; orally administered non-absorbable antibiotics were effective in eradicating CRE colonization in a significantly higher proportion of treated patients compared to controls, providing the first prospective controlled evidence in a predominantly hematological cohort (Oren et al., 2013). These findings were further supported by Lambelet et al., who treated 14 HM patients with oral gentamicin for carbapenem-resistant *K. pneumoniae* gut colonization and reported an overall decontamination rate of 71%, rising to 90% among patients not receiving concomitant systemic antibiotic therapy, with no new gentamicin-resistant isolates detected during treatment (Lambelet et al., 2017). The only RCT specifically designed for this population was conducted by Stoma et al. in a tertiary hematology center, evaluating oral colistin over 14 days in adult patients with HM colonized by MDR/XDR Gram-negative bacteria (mostly Enterobacterales): a statistically significant short-term decolonization benefit was demonstrated at day 14 (61.3% vs 32.3%; OR 3.32; 95% CI 1.17–9.44; p=0.024), with a lower incidence of bloodstream infections in the intervention arm within the first 30 days (3.2% vs 12.9%); however, no sustained advantage was observed at day 21 post-treatment or over a 90-day follow-up, and no serious adverse effects or increase in colistin resistance were detected (Stoma et al., 2018). Taken together, the available evidence suggests that oral SDD, primarily with gentamicin or colistin, selected according to individual susceptibility profiles, may reduce intestinal CRE burden in HM patients, with a potential short-term reduction in BSI rates; however, the durability of decolonization is consistently limited, the risk of secondary resistance emergence is a recognized concern, and no strategy has demonstrated a sustained impact on long-term infection rates or overall survival in adequately powered RCTs.

Several studies currently indexed on ClinicalTrials.gov (Manges, 2019; Rambam Health Care Campus, 2020; DAVIDO, 2021; Microbiome Health Research Institute, 2021; Zerr, 2021; Liu et al., 2022; Kraft, 2023; Lee, 2023; FMT for Multidrug Resistant Organism Reversal, n.d.) have shown that FMT is the most promising approach in this patient population.

Although the main indication for FMT in adults is CDI, followed by gut graft-versus-host disease (GVHD), FMT procedures have also been successfully performed for microbiota restoration in AML and the eradication of MDRO in both children and adults diagnosed with hematological diseases (Freedman and Eppes, 2014; Merli et al., 2020, 2022; Battipaglia et al., 2023, 2023).

A case report of CRE colonization in an adult patient after allogeneic hematopoietic stem cell transplantation (allo-HSCT) with recurrent CRE infections demonstrated successful eradication with FMT (Su et al., 2021). Battipaglia et al. conducted a retrospective single-center study of patients with hematologic malignancies undergoing FMT before or after allo-HSCT for MDRB colonization or infection. Decolonization occurred in 7/10 patients; persistent decolonization at last follow-up was in 6/10 patients (Battipaglia et al., 2019). Bilinski and colleagues conducted an uncontrolled study of 20 adult HM patients colonized by CRE, ESBL-E, or other MDRO, finding that FMT was effective in eradicating 60% of MDROs at 1 month and 93% at 6 months (Bilinski et al., 2017). Twenty-two pediatric HSCT recipients (age range 0.8-18.5 years) who were concomitantly colonized by CRE have also been reported to undergo FMT before or after HSCT to decolonize them from CRE. The decolonization rate was 77% and 52% at 1 and 6 weeks post-FMT, respectively. All patients decolonized at 6 weeks had at least 3 consecutive negative samples, taken 1 week apart (Masetti et al., 2025). A detailed overview of the described studies, including patient characteristics, decolonization strategies, and clinical outcomes, is provided in **Table 1**.

However, during FMT, resistance genes can be acquired, especially in immunocompromised subjects (Leung et al., 2018). In 2019, the Food and Drug Administration (FDA) reported two cases of immunocompromised adults (one of whom died) who developed invasive infections caused by ESBL-Ec after receiving experimental FMT and issued a safety alert requiring screening of donors for MDRO (Manges, 2019; Rambam Health Care Campus, 2020; DAVIDO, 2021; Microbiome Health Research Institute, 2021; Zerr, 2021; Lui, 2022; Kraft, 2023; Lee, 2023; FMT for Multidrug Resistant Organism Reversal, n.d).

Alternative approaches, including probiotics and bacteriophage therapy, currently lack evidence specifically in this population and remain at early preclinical or proof-of-concept stages. Therefore, well-designed, multicenter randomized trials are urgently needed to establish effective and durable decolonization protocols for CRE-colonized HM patients. However, future microbiome-directed interventions should aim to restore the ecological and immunological functions of the intestinal ecosystem. Re-establishing microbial diversity, promoting immune reconstitution at the mucosal interface, and recovering colonization resistance may collectively reduce intestinal CRE persistence and limit bacterial translocation, ultimately decreasing the risk of CRE-associated bloodstream infections in this highly vulnerable patient population.

## 5 Conclusions

**Figure 3:**
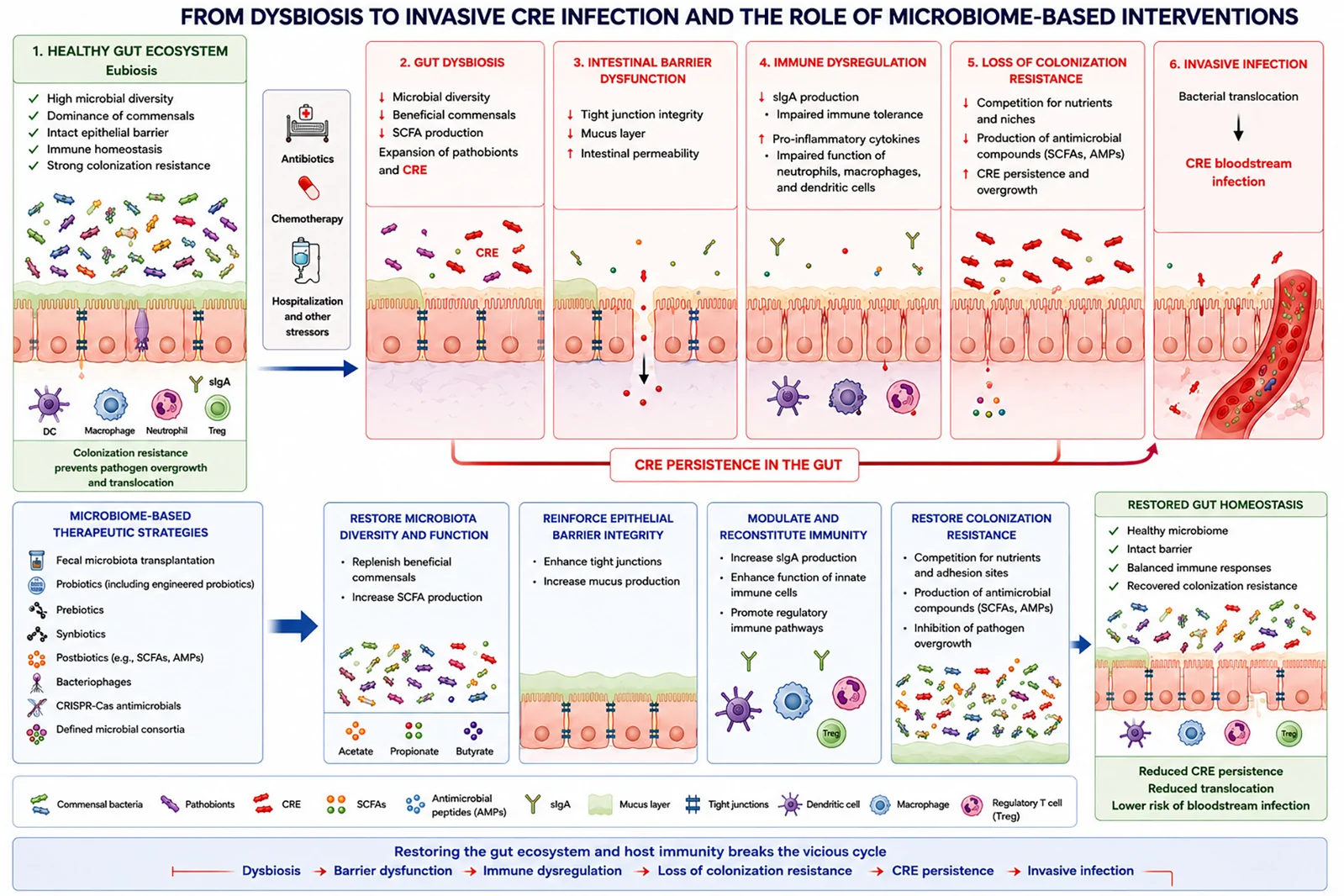
Pathophysiological progression from gut eubiosis to gut dysbiosis and invasive CRE infections (top panel). The restorative mechanisms of microbiome-based interventions are illustrated (bottom panel).

The aim of this review was to provide an overview of CRE carriage and infections, related GM alterations, and cutting-edge strategies currently being explored to eradicate these pathogens from the GM, in both the general population and HM patients. All of the aforementioned approaches are still in preclinical or clinical development or in the approval stage. Currently, the scientific community lacks sufficient data, necessitating further, more in-depth studies to overcome the limitations of these methodological approaches and, above all, to obtain definitive results on efficacy and safety sufficient to establish official use and recommendations. However, despite current doubts about the potential applicability of each of these strategies, they show very promising results to date, and the shared goal is to successfully integrate these methodologies into common clinical practice for modulating the GM for decolonization of CRE, particularly in fragile and delicate patients, such as HM patients. Future efforts are expected to progressively shift toward precision microbiome therapeutics, including engineered probiotics, defined microbial consortia, postbiotics, and personalized microbiome-guided interventions (**Figure 3**). Moreover, integrating multi-omics approaches, such as metagenomics, metabolomics, and resistome profiling, may enable more accurate identification of patients at risk of persistent CRE colonization and support the development of more targeted and effective microbiome-based therapies.
